# Pulmonary alveolar proteinosis in children: a case series

**Published:** 2010

**Authors:** Seyyed Ahmad Tabatabaei, Abdollah Karimi, Sedigheh Rafiee Tabatabaei, Badiozzaman Radpay, Farzaneh Jadali, Farideh Shiva, Mana Hadipour Jahromy

**Affiliations:** a Assistant Professor of Pediatrics, Pediatric Pulmonary Fellowship, Mofid Children Hospital, Shahid Beheshti Medical University, Tehran, Iran; b Professor of Pediatric Infectious Diseases, Pediatric Infectious Research Center, Mofid Children Hospital, Shahid Beheshti Medical University, Tehran, Iran; c Associate Professor of Pediatrics, Pediatric Infectious Research Center, Mofid Children Hospital, Shahid Beheshti Medical University, Tehran, Iran; d Associate Professor of Anesthesiology, Masih Daneshvari Hospital, Shahid Beheshti Medical University, Tehran, Iran; e Associate Professor of Pathology, Pediatric Infections Research Center, Mofid Children Hospital, Shahid Beheshti University of Medical Sciences, Tehran, Iran; f General Practitioner, Researcher, Pediatric Infections Research Center, Shahid Beheshti University, Tehran, Iran

**Keywords:** Pulmonary Alveolar Proteinosis, Respiratory Failure, Respiratory Distress, Children

## Abstract

Pulmonary alveolar proteinosis, (PAP) is a rare disease of unknown etiology, characterized by accumulation of intraalveolar proteinaceous material which is rich in lipid and positive on periodic acid-Schiff stain. Two clinically different pediatric types have been defined as congenital PAP which is fulminant and fatal, and a late-onset PAP which is similar to the adult form and less severe. Eight children with late-onset PAP were hospitalized from 1998 to 2005 in Mofid Children Hospital. Characteristics of these patients and the methods of diagnosis and treatment are presented in this case series.

Pulmonary Alveolar Proteinosis (PAP) first described by Rosen et al in 1958.^1^ PAP is a rare disease of unknown etiology, characterized by the accumulation of proteinoceous material in the alveoli that is rich in lipid and is positive on periodic acid-Schiff (PAS) stain.^2^–^4^ Although its exact pathogenesis is unclear, most investigators have postulated a decreased clearance of surfactant from the air spaces.^4^,^5^

PAP is rare in children, with only a few dozen cases reported in the literature.^2^ Today, PAP can be categorized in to three different classes: congenital, acquired, and secondary to other conditions, such as malignancy (i.e. leukemia and lymphoma) and immunodeficiency.^6^,^7^ Two forms are encountered in pediatric practice: congenital alveolar proteinosis (CAP) and a later-onset form that is generally less severe. These 2 types differ in respect to etiology, clinical course, therapy and outcome.^3^ Family history of a similarly affected sibling or consanguinity suggests autosomal recessive transmission.^2^ The later-onset form always appears after a postnatal, symptom-free period ranging from a few weeks to several years. The main symptoms are non-specific, including progressive onset dyspnea during feeding or exercise and then at rest, cough, cyanosis and digital clubbing. Asthenia and growth retardation are common. The progression of the disease is very variable, ranging from asymptomatic forms diagnosed by chance to earlyonset forms that progress rapidly and result in uncontrollable respiratory failure.^2^,^8^

The classic radiologic appearance of PAP is bilateral, symmetric, perihilar airspace consolidation in a bat-wing distribution.^3^

The diagnosis of postnatal onset pediatric PAP is based on examination of the BAL sample and/or lung biopsy.^2^ Broncho-alveolar lavage is the key to diagnosis.^2^,^5^,^9^

The most effective treatment for PAP is the mechanical removal of the proteinaceous material via whole lung lavage (WLL)^10^–^12^ which was initially proposed by Ramirez in 1965, this method is still the only therapy that is really effective. Other therapeutic trials have been proposed such as: lung transplantation, adminstration of GM-CSF and gene-therapy.^2^,^3^,^13^ This report describes eight cases of PAP which were diagnosed by open lung biopsy in Mofid Children Hospital in Tehran.

## Case Report

The main characteristics of patients are presented in table 1. The median presentation age was 2.4 years, (6 mo-5 yr). Consanguinity was found in five patients.

**Table 1:** The main characteristics of seven children with PAP, Mofid Children Hospital, Tehran

No	Gender	Age of onset	Age of Dx	Main symptoms	Main signs	Consanguinity	Hemoglubin	PaO_2_mm Hg	Radiologic findings	Dx tools	Treatment	Last follow up after Dx	Outcome
1	F	2.5 y	3 y	Dry cough fever FTT*	Tachypnea/fine rales	+	**15.5**	45.8	Diffuse alveolar infiltrates	OLB***	GM-CSF†	9 mo	Expired at home	
2	F	2.5 y	4.5 y	Dry cough FTT exercise intolerance	RD** diminished breath-soundschest muscle retraction	+	**10.2**	49	Diffuse alveolar infiltrates/airbroncho gram	OLB	WLL†† × 2 with CPB†††	8 mo	Expired due to pulmonary hemorrhage after lavage	
3	M	3 y	4 y	Dry cough cyanotic attacks anorexia exercise intolerance	RD diffuse crackles/ sys heart murmur	−	**12.3**	60	Diffuse alveolar infiltrates/airbroncho gram/hilar adenopathy	OLB	WLL × 2 with CPB	15 mo	Expired at home
4	M	3.5 y	4 y	Dry cough cyanosis exercise intolerance	Cyanosis RD diffuse crackles	+	**12**	50.5	Diffuse alveolar infiltrates/airbroncho gram	OLB	GM-CSF / segmentai & WLL with CPB & ECMO §	36 mo	O2 DEP§§ alive	
5	M	6 mo	6 mon	Dry cough poor feeding exercise intolerance	Diffuse crackles RD chest muscle retraction	−	**8.9**	61.9	Diffuse alveolar infiltrates/ground glass appearance	OLB	Antibiotics + O_2_ therapy	1 mo	Expired after OLB
6	M	2.5 y	4 y	Severe cyanosis progressive apnea exercise intolerance	Severe cyanosis RD respiratory arrest	+	**12**	−	Diffuse alveolar infiltrates	OLB	O_2_ therapy/WLL × 2 with CPB	36 mo	Free of O_2_alive
7	F	4 y	5 y	Dry cough anorexia FTT exercise intolerance	Tachypnea RD rales on left lung	UN	**11**	60 with	Diffuse alveolar infiltrates/hilar adenopathy	OLB	WLL × 3	6 mo	Expired after third lavage
8	F	1 y	1 y	FTT cough cyanosis	Tachypnea RD	+	**14.4**	52	Diffuse alveolar infiltrates/ air bronchogram	OLB	−	1 y	Expired due to RD after OLB	

* FTT: Failure to thrive;

** RD: Respiratory distress;

*** OLB: Open lung biopsy;

† GM-CSF: Granulocyte macrophage colony stimulating factor;

†† WLL: Whole lung lavage;

††† CPB: Cardio pulmonary bypass;

§ ECMO: Extracorporeal membrane oxygenation;

§§ O_2_ DEP: Oxygen dependent

**Figure 1:**
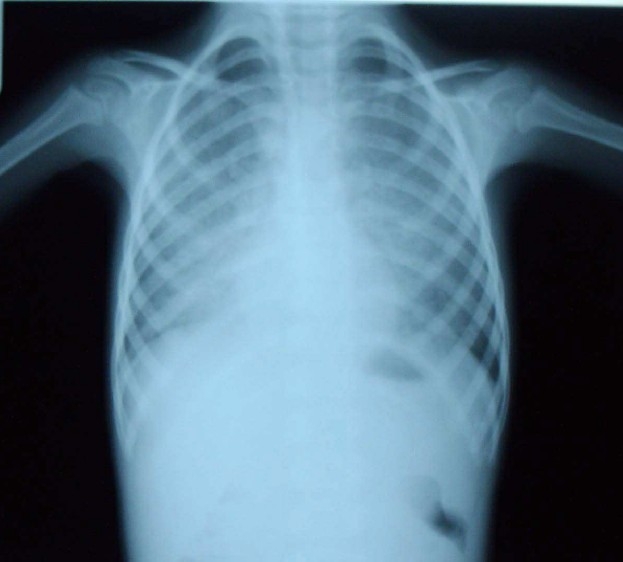
Chest X-ray of pulmonary alveolar proteinosis in patient no. 4

**Figure 2:**
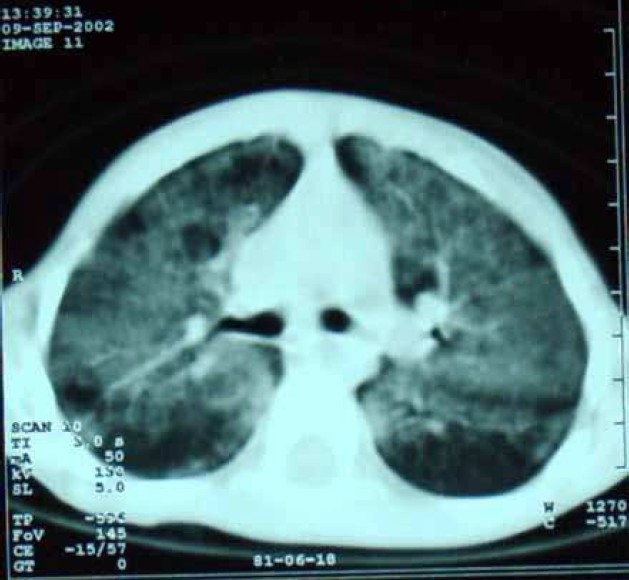
Chest CT-scan of patient no. 4 with pulmonary alveolar proteinosis

**Figure 3:**
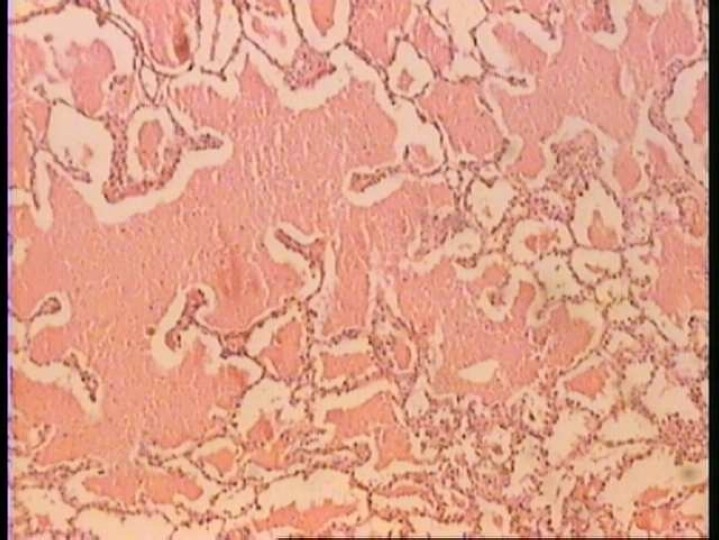
Pathologic feature of PAP in patient no. 6: presence of marked diffused intraalveolar pinkish deposition; positive with PAS staining. Serial sections were reviewed and no inflammatory infiltrate, parasite and fungi were identified.

The main presenting symptoms were growth retardation, exercise intolerance, cough, progressive dyspnea and oxygen dependency. Chest X-ray revealed diffuse alveolar infiltration in seven children with an air bronchogram in four (Figure 1). Echocardiography revealed no anatomical shunt but RA and RV dilatation, TR, dilated PA and high cardiac output were noted in 5 patients. Echocardiography was normal in two children. Chest CT scan was done in three patients, which showed diffuse interstitial and alveolar infiltration (Figure 2). Open lung biopsy (OLB) confirmed the diagnosis in all children. Figure 3 shows the pathologic feature of case no. 6. Secondary causes of PAP were ruled out by history taking and lab tests but checking anti-GMCSF antibody or gene mutation were unavailable.

For two first patients GM-CSF was started subcutaneously because WLL was not practically available in present setting at first. First patient received it for 3 months, and the parents didn’t continue it, and she died after 6 months. For the second patient (case no. 4) GMCSF was used for 2 months. The response monitoring was done by clinic (cyanosis, respiratory rate, activity, and well-being), oxygenation (SpO_2_and ABG) and X-ray. None of these changed during the administration of GMCSF.

Lobar or segmental lavage by fiberoptic bronchoscope is useful when PAP is less advanced or general anesthesia is hazardous.^4^ This method could be done in most hospitals and proteinaceous fluid can be removed with a small volume of saline.^4^ The segmental lung lavage was tried for PAP with fiberoptic bronchoscope. Only 1-2 segments in each procedure could be lavaged. It was repeated for 3 times and it was partially effective. Therapeutic whole lung lavage (WLL) is carried out under general anesthesia and each time one lung, lobe or segment is washed with normal saline^2^,^4^; finally both lungs were washed while patients were on CPB; using ECMO didn’t facilitate the WLL. The procedure was done in five patients and due to recurrence, repeated WLL was necessary in 4 patients. Two patients with WLL are alive after 5 years, and one of them is free of oxygen. Six patients died, one due to pulmonary hemorrhage during WLL and five within 0.5-15 months after the diagnosis due to severe respiratory failure.

## Discussion

Clinical presentation with cough and progressive dyspnea in present cases was compatible with other case series^1^–^16^; though, chest pain, hemoptysis, fever and weight loss have also been reported.^5^,^15^,^17^

Due to the rarity of PAP, non-specific signs and symptoms, variability in severity and course, and the fact that it may be asymptomatic in up to 30% of cases, the diagnosis can be easily delayed or missed.^5^,^8^ There was an average of 9.7 month delay between presentation and diagnosis in present patients.

The radiologic findings in present patients showing diffuse alveolar infiltration were consistent with prior studies, although interstitial and nodular patterns have been reported.^3^,^15^

Although BAL examination can be sufficient for diagnosis of PAP in adults, but in children, the histopathologic examination of open transthorasic or transbronchial lung biopsy is the gold standard for diagnosis.^4^ Unfortunately checking anti-GMCSF antibody and measuring surfactant protein B were unavailable, and to rule out other similar diseases open lung biopsy was preferred to confirm PAP.

In this study, time from beginning of clinical manifestations to diagnosis was approximately 11 months; this delay may be due to non-specific clinical and radiological findings, variable progression course and physician’s unfamiliarity with the disease.

Although administration of GM-CSF may improve pulmonary function in adults and it may be effective in children, the first 2 patients who took it empirically did not improve.^9^ It is believed that mechanical removal of proteinaceous material (WLL) by an expert team may be the most suitable therapy in Iran especially in children, as it improved the clinical course in four out of five of patients. Because the natural course of the disease may be recurrent, WLL may be needed repeatedly. In a pediatric case study the WLL patients did well,^18^ but 3 of present cases died, one during the procedure due to pulmonary hemorrhage and 2 succumbed after repeated WLL due to recurrence.

Mortality rate was 75% in present cases, similar to other reports with the duration of illness ranging from a few days to several months. The mortality of congenital PAP is 100% without lung transplantation.^3^ Late-onset disease has better prognosis, but repeated lung lavage is generally required.

It is believed that the poor prognosis in present patients is due to delay in diagnosis, severity of disease, non-compliance of family for recurrent WLL and in a few patients, lack of close team collaboration.

## Conclusions

In conclusion, the authors emphasize that missed and delayed diagnosis could be avoided by physicians’ awareness of PAP; diagnosis of postnatal onset PAP should be considered in every patient with respiratory distress or failure with diffuse interstitial and alveolar infiltration.

Examination of the BAL sample and/or a lung biopsy is the key to diagnosis and atypical specimens should be approached with caution which may represent either PAP or other pulmonary diseases with secondary accumulation of surfactant WLL could be the most suitable treatment for late-onset PAP in Iran.
